# The Wako-Saitô-Muñoz-Eaton Model for Predicting Protein Folding and Dynamics

**DOI:** 10.3390/molecules27144460

**Published:** 2022-07-12

**Authors:** Koji Ooka, Runjing Liu, Munehito Arai

**Affiliations:** 1 Department of Physics, Graduate School of Science, The University of Tokyo, 3-8-1 Komaba, Meguro, Tokyo 153-8902, Japan; ooka-koji063@g.ecc.u-tokyo.ac.jp; 2 Komaba Organization for Educational Excellence, College of Arts and Sciences, The University of Tokyo, 3-8-1 Komaba, Meguro, Tokyo 153-8902, Japan; 3 Department of Life Sciences, Graduate School of Arts and Sciences, The University of Tokyo, 3-8-1 Komaba, Meguro, Tokyo 153-8902, Japan; liurunjing@g.ecc.u-tokyo.ac.jp

**Keywords:** protein folding, statistical mechanical model, WSME model, folding kinetics, folding intermediates, protein dynamics

## Abstract

Despite the recent advances in the prediction of protein structures by deep neutral networks, the elucidation of protein-folding mechanisms remains challenging. A promising theory for describing protein folding is a coarse-grained statistical mechanical model called the Wako-Saitô-Muñoz-Eaton (WSME) model. The model can calculate the free-energy landscapes of proteins based on a three-dimensional structure with low computational complexity, thereby providing a comprehensive understanding of the folding pathways and the structure and stability of the intermediates and transition states involved in the folding reaction. In this review, we summarize previous and recent studies on protein folding and dynamics performed using the WSME model and discuss future challenges and prospects. The WSME model successfully predicted the folding mechanisms of small single-domain proteins and the effects of amino-acid substitutions on protein stability and folding in a manner that was consistent with experimental results. Furthermore, extended versions of the WSME model were applied to predict the folding mechanisms of multi-domain proteins and the conformational changes associated with protein function. Thus, the WSME model may contribute significantly to solving the protein-folding problem and is expected to be useful for predicting protein folding, stability, and dynamics in basic research and in industrial and medical applications.

## 1. Introduction

Many proteins fold into specific three-dimensional (3D) structures to perform their functions and drive various biological processes. Therefore, elucidating how proteins fold is essential for understanding the fundamental processes of life. Since the existence of protein-folding pathways was first proposed [1], the detection and characterization of intermediates and transition states during folding reactions have been extensively performed using a variety of experimental techniques [2,3,4,5]. Theoretical studies of protein-folding reactions, including molecular-dynamics (MD) simulations of all-atom models and Monte Carlo simulations of coarse-grained models, have made significant progress in explaining experimental observations [6,7,8,9,10,11,12]. In particular, statistical mechanical approaches have shown that protein-folding processes can be comprehensively described by the free-energy landscapes of proteins [7,8,9,13,14,15,16].

The Wako–Saitô–Muñoz–Eaton (WSME) model is promising for describing protein-folding reactions [17]. The WSME model is a coarse-grained model of proteins based on a simple and elementary statistical mechanical theory and can readily calculate free-energy landscapes using the 3D native structures of proteins [13,18,19,20]. The free-energy landscapes obtained by the WSME model comprehensively predict both the thermodynamic stability of proteins under equilibrium conditions and their kinetic folding processes under non-equilibrium conditions, including folding pathways, folding-rate constants, and the structures of the intermediates and transition states. The predictions were consistent with the experimental results in many cases, especially for the folding of small single-domain proteins [13]. To date, many extensions and modifications have been implemented in the model to accommodate a variety of experimental conditions, contributing significantly to our understanding of protein-folding mechanisms. Furthermore, theoretical predictions by the WSME model play an important role in complementing MD simulations, resolving discrepancies between simulations and experiments, and bridging the gap between them. The WSME model has also been used to estimate the effects of amino-acid substitutions on proteins and to explain conformational changes accompanied by protein function, making it a promising protein-engineering tool for industrial and medical applications [21,22]. Because protein folding and dynamics have been extensively studied using the WSME model, it would be useful to summarize the previous progress and discuss the issues that remain to be resolved.

In this review, we first describe the details of the basic WSME model and outline how to calculate the free-energy landscapes of proteins in Section 2. The subsequent sections summarize the applications of the WSME model for predicting the folding processes of small single-domain proteins (Section 3) and of multi-domain proteins with complex folding mechanisms (Section 4). Section 5 presents extended versions of the WSME model for analyzing the protein dynamics observed in intrinsically disordered proteins, functional motions, and amyloid fibril formation. Finally, the future challenges and prospects of the WSME model are discussed in Section 6.

## 2. WSME Model

### 2.1. Description of the Model

The WSME model is a coarse-grained statistical-mechanical model based on the 3D structures of proteins. In 1978, Wako and Saitô originally proposed the basic ideas and calculation methods of this model (called the “island model”) [18,19]. Approximately 20 years later, Muñoz and Eaton rediscovered it in 1999 [13]. Since then, this model has been termed the Wako–Saitô–Muñoz–Eaton (WSME) model. The WSME model is a Gō-type model that considers only the interactions formed in the native state of proteins without considering non-native interactions [23]. Gō proposed the consistency principle, which holds for ideal proteins and states that the most stable structure of a local fragment taken from a protein is consistent with the native structure of the full-length protein. In other words, the interactions that stabilize the local structure of a protein are consistent with the interactions that stabilize the overall structure of a protein [24]. Such ideal proteins can be virtually constructed by considering only the interactions that stabilize the native structure; this type of potential is called the Gō potential [25]. The WSME model uses the Gō potential, assuming the consistency principle, and describes the folding and dynamics of ideal proteins. The consistency principle is considered equivalent to the principle of minimal frustration, which states that frustration in energy arising from stabilization by non-native contacts is minimized in foldable proteins [7,16]. Therefore, the WSME model also assumes the principle of minimal frustration. Due to its simplicity, the WSME model can readily calculate the free-energy landscape of protein folding.

**Figure 1:**
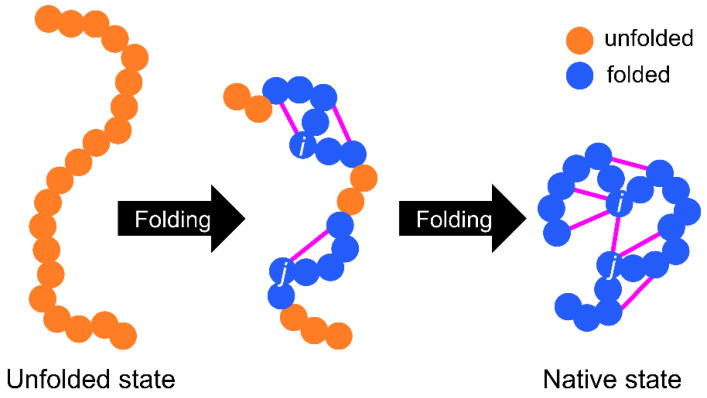
Schematic representation of a protein-folding process in the Wako–Saitô–Muñoz–Eaton (WSME) model. Residues in folded or unfolded conformations are indicated by blue and orange circles, respectively. Native contacts indicated by magenta lines are formed only when all intervening residues are cooperatively folded into the native-like conformations.

**Figure 2:**
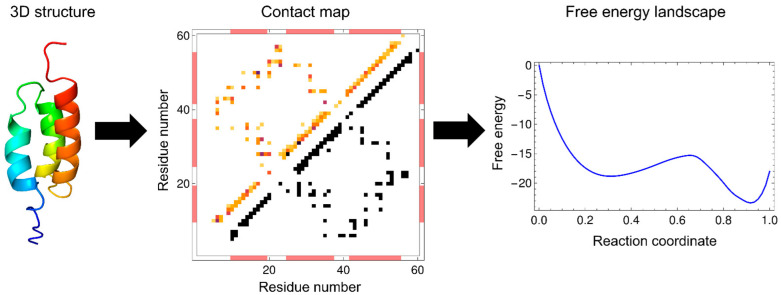
Basic procedure for calculating free energy with the WSME model. Based on the protein three-dimensional structure (left panel: the B domain of protein A (BdpA); PDB ID: 1BDD), a residue–residue contact map of the protein is calculated (middle panel). A pair of *i*- and *j*-th residues (*j* > *i* + 2) is defined as being in contact when at least one of the distances between the atoms in the *i*-th residue and those in the *j*-th residue is less than 4 Å in the native state. The triangle in the lower right half (with black squares) is a binary contact map with a uniform contact energy, and the triangle in the upper left half (with colored squares) is a non-binary contact map weighted by the number of atoms in the native contacts. The partition function is calculated from the Hamiltonian based on the contact map, and the free energy is obtained as a function of the reaction coordinate (right panel). The one-dimensional free-energy landscape calculated using the uniform contact energy is shown. Adapted with permission from Ref. [26]. Copyright (2006) National Academy of Sciences, USA.

The basic WSME model is as follows (Figure 1 and Figure 2). First, an Ising-like two-state variable *m_k_* is assigned to each residue of a protein. The index *k* represents the residue number. *m_k_* is 1 when the residue is in the native-like conformation and 0 when the residue is in other conformations. The protein state {*m*} is defined as a set of residue states (*m*_1_, *m*_2_, *…*, *m_N_*), where *N* is the total number of residues. Next, the protein state has 2*^N^* possible conformations. The Hamiltonian of the WSME model is defined as:

(1) $$H(\left\{m\right\})=\sum_{i=1}^{N-1}\sum_{j=i+1}^{N}\varepsilon_{i,j}\Delta_{i,j}m_{i,j},$$

where Δ*_i,j_* represents the native contact between residues; Δ*_i,j_* = 1 when *i*- and *j*-th residues are in contact with each other in the native state, otherwise Δ*_i,j_* = 0 (Figure 1). *ε_i,j_* is the contact energy between *i*- and *j*-th residues in the native state and takes negative values when a stable interaction is formed in the native state (Figure 2). *m_i,j_* is defined as:

(2) $$m_{i,j}=m_{i}m_{i+1}\cdots m_{j}=\prod_{k=i}^{j}m_{k},$$

and *m_i,j_* = 1 only when all residues between *i*- and *j*-th residues are in native-like conformations. This implies that native interactions between *i*- and *j*-th residues are established only when all intervening residues are cooperatively folded into their native conformations (Figure 1). Therefore, this model assumes that folding is initiated by local interactions between neighboring residues and spreads to distal regions via the growth and docking of native segments (Figure 1). The number of states *W* is defined as follows:

(3) $$W(\{m\})=\exp\left[\left(S_{0}+\sum_{i=1}^{N}S_{i}m_{i}\right)/k_{B}\right],$$

where *k*_B_ is the Boltzmann constant, *S*_0_ is the conformational entropy of the fully unfolded state, and *S_i_* (<0) is the entropic reduction attributed to the formation of the native conformation. Then, the partition function is described as:

(4) $$\begin{array}{ll} Z & =\sum_{\mathrm{All}\;\mathrm{states}}W(\{m\})\exp\left[-\frac{H(\{m\})}{k_{B}T}\right]\\ & =\sum_{\mathrm{All}\;\mathrm{states}}\exp\left[-\frac{1}{k_{B}T}\left(\sum_{i=1}^{N-1}\sum_{j=i+1}^{N}\varepsilon_{i,j}\Delta_{i,j}m_{i,j}-T\sum_{i=1}^{N}S_{i}m_{i}\right)\right]. \end{array}$$

*S*_0_ is neglected in Equation (4) because it is a constant and does not affect the results of the free-energy calculation. Thus, the effective free energy of a native stretch from *i*- to *j*-th residue can be described as:

(5) $$F_{i,j}=\sum_{k=i}^{j-1}\sum_{l=k+1}^{j}\varepsilon_{k,l}-T\sum_{k=i}^{j}S_{k},$$

where the first and second terms are enthalpy and entropy, respectively. Equation (5) shows that the progress of the folding reaction is enthalpically favorable due to the formation of native interactions, but is entropically unfavorable due to the reduction in the number of possible states. Such enthalpy–entropy compensation is directly reflected in the WSME model, and the balance between them results in free-energy barriers. The extent to which protein folding proceeds is often used as a reaction coordinate, such as the fraction of residues in the native state, $n=\sum_{i=1}^{N}m_{i}/N$, and the fraction of native contacts formed, $Q=\sum_{i<j}^{}\Delta_{i,j}m_{i,j}/\sum_{i<j}^{}\Delta_{i,j}$. The WSME model can calculate the free energy from the partition function restricted to the value of the reaction coordinate (Figure 2).

The WSME model was originally developed by Wako and Saitô in 1978 to study the statistical mechanical properties of ideal biopolymers, including proteins [18,19]. Subsequently, the model was applied to predict pathways and intermediates in the folding of several proteins by calculating the free-energy landscapes and residue-specific structure formation along reaction coordinates [27]. In the 1980s, the idea underlying the WSME model, that native contacts are formed in local segments (islands) and that native islands grow into entire proteins, was applied to two-dimensional (2D) and 3D lattice models of protein folding [23,24,28] and to a Potts-like model with three states (α-helix, β-strand, and coil) [29], showing that this idea is useful for describing the nature of protein-folding transitions. In the 1980s and 1990s, experimental data characterizing detailed protein-folding reactions increased, especially through the use of Φ-value analysis to investigate structure formation in the transition states [30,31,32], and through the use of the pulsed-hydrogen exchange nuclear magnetic resonance (NMR) technique to examine the structures of kinetic intermediates [33,34,35,36]. This prompted the need for theoretical models to explain the experimental results. In 1999, Muñoz and Eaton rediscovered the WSME model and succeeded in predicting the free-energy landscapes and folding rates for 18 small proteins that folded in a two-state manner; the findings were in good agreement with the experimental data [13]. These results indicated that the WSME model is promising for explaining the experimental results of protein folding. Furthermore, the results suggest that real small proteins can be approximated by ideal proteins that satisfy the consistency principle and principle of minimal frustration.

### 2.2. Calculation of the Partition Function

**Figure 3:**
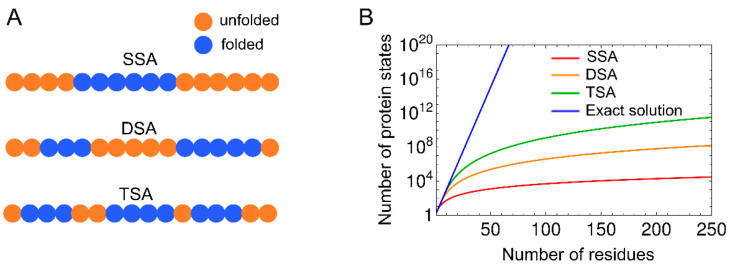
(**A**) Schematic representation of protein states with single, double, and triple sequence approximations (SSA, DSA, and TSA, respectively). Residues in folded or unfolded conformations are indicated by blue and orange circles, respectively. In the SSA, only the protein states with a single native segment are considered. In DSA and TSA, the protein states with up to two and three native segments are considered, respectively. (**B**) Number of microscopic protein states, which is considered in the calculation of the partition function using the SSA, DSA, TSA, and exact solution, is plotted against the number of residues.

There are several methods for calculating the partition function of the basic WSME model and its variants. Since the number of states for an *N*-residue protein is 2*^N^* in the WSME model, and the computational complexity increases exponentially with increasing protein size, it is impossible to numerically calculate the partition function using Equation (4), even for a protein with ~50 residues. Thus, approximations that consider only specific protein states along the folding reaction coordinates are sometimes used. For example, single, double, and triple sequence approximations (SSA, DSA, and TSA) assume that up to one, two, and three independently folded segments, respectively, are allowed during the folding process (Figure 3) [13,37]. These approximations reduce the number of states to a polynomial quantity, enabling the calculation of the partition function, even for proteins with ~100 residues. DSA with loops (DSA/L), a variant of DSA, was developed, which involves non-local interactions between two folded segments [38,39,40,41,42,43,44]. DSA/L predicts fewer cooperative folding transitions than the original model because of the presence of non-local interactions. Non-local interactions play an essential role in the folding of large proteins because interactions between distant regions can affect their folding processes. In line with this, the introduction of non-local interactions between the N- and C-termini by a virtual linker in a multi-domain protein successfully explained folding processes that were not explained by the original model [45]. In addition, mean-field approximation was discussed for computing the partition function of the WSME model [46,47].

An exact solution to the WSME model using transfer matrices was reported by Bruscolini and Pelizzola in 2002 [20]. The exact solution enables efficient calculation of the partition function by summing all 2*^N^* states (Figure 3), and the calculation, even for large proteins with ~200 residues, is completed instantaneously.

Comparison of the approximate solutions, such as SSA, DSA, DSA/L, and TSA, with the exact solution of the WSME model was performed by calculating the free-energy profiles and folding rates for many proteins [37]. SSA did not describe reasonable free-energy barriers on one-dimensional (1D) free-energy landscapes because it is a coarse sampling method and considers only a single folded segment [37]. By contrast, all calculation methods other than SSA were able to predict folding rates, which was consistent with experimental results. The calculated free-energy profiles were almost unchanged, irrespective of the reaction coordinates used (*n* or *Q*). The finding that the calculations with DSA and TSA yielded similar results to the exact calculation suggests that the number of native segments formed during the folding process is 2 or 3 for small proteins. This was also shown in long-duration all-atom MD simulations, confirming that simple assumptions, such as DSA/L, are sufficient to explain the folding mechanisms of small proteins [42]. Note that for small proteins with fewer than ~50 residues, calculations with the exact solution overestimated the cooperativity of folding transitions, whereas those with DSA/L were the most consistent with the experiments [42,43].

### 2.3. Contact Energy

Calculation of the free energy using the WSME model requires setting an entropic cost associated with the folding reaction and preparing a residue–residue contact map and the energy for each contact based on the native structure of a protein (Figure 2). A residue pair is defined as being in contact when the distance between two residues in the native state is within a specific cutoff (typically 4 Å). The simplest way to assign contact energies is to use the same energy value for all the native contacts. Surprisingly, even with this simple treatment, the calculated free-energy landscapes effectively explained the folding processes experimentally observed for many small proteins [26,37,39,40,41,42,43,48,49]. Another way of assigning contact energies is to weigh the contact energy, depending on the number of atoms involved in the contact [13,20,45,50,51,52,53]. The use of weighted contact energies may yield more accurate results than the use of uniform contact energies.

Interestingly, sequence-dependent weighting of contact energies is not always necessary to describe the folding pathway for some small proteins [43]. Using DSA/L, Kubelka et al. investigated the effects of contact energy weighting on the free-energy landscapes for two proteins with a helix-turn-helix motif (P22 subdomain and αtα). They compared three contact energies: those weighted by the structural ensemble determined by NMR, those statistically determined by Miyazawa and Jernigan, and sequence-independent uniform contact energies. The results showed that the use of uniform contact energies was sufficient to explain the folding processes and to predict different folding pathways for two proteins with the same fold, reflecting subtle differences in local stability [43]. This suggests that the main-chain structure (i.e., protein topology or fold) itself contains sufficient information about the folding processes, and that the assumptions in the WSME (DSA/L) model successfully decode the folding mechanisms encoded in the contact map of the native structure.

Because homologous proteins with similar topologies have similar contact maps, uniform contact energies predict similar folding mechanisms for homologous proteins. However, even proteins with the same fold may have different folding processes, depending on the amino-acid sequence [54,55,56,57,58,59,60]. Such differences in the folding mechanisms cannot be distinguished by calculations using uniform contact energies. Therefore, obtaining suitable contact energies to describe the details of folding mechanisms is a challenge in optimizing the WSME model. Several studies have attempted to evaluate residue–residue contacts by considering the contribution of non-covalent interactions that drive protein folding, including electrostatic interactions, hydrogen bonds, van der Waals interactions, and hydrophobic interactions [61,62,63,64]. In addition, the introduction of temperature-dependent enthalpy and entropy terms [65,66] and the calculation of contact energies using AMBER force fields [67], which are typically used for MD simulations, have also been proposed. Such rigorous contact evaluations based on physical chemistry allow the calculation of contact energies for all residue pairs, rather than selecting residue–residue contacts according to an inter-residue distance cutoff. These approaches may require the determination of additional parameters, such as scaling constants, in order for predictions to agree with experimental results, including the temperature-dependent denaturation curves monitored by circular dichroism or NMR spectroscopy [43,53,68,69], the temperature dependence of specific heat capacity [40,41,63,65,69,70,71,72,73], and the denaturant dependence of folding/unfolding rate constants (called a chevron plot) [38,74].

## 3. Prediction of Folding Mechanisms

### 3.1. One-Dimensional Free-Energy Landscape: Two-State Versus Downhill Folding

The 1D free-energy landscape obtained using the WSME model is a powerful tool for analyzing protein stability under equilibrium conditions. Once the free-energy landscape is calculated, metastable intermediates and free-energy barriers can be clearly visualized, and folding mechanisms can be directly analyzed from the free-energy surface. Furthermore, the WSME model provides an effective analytical method for investigating the temperature and denaturant dependence of folding pathways [67,75].

**Figure 4:**
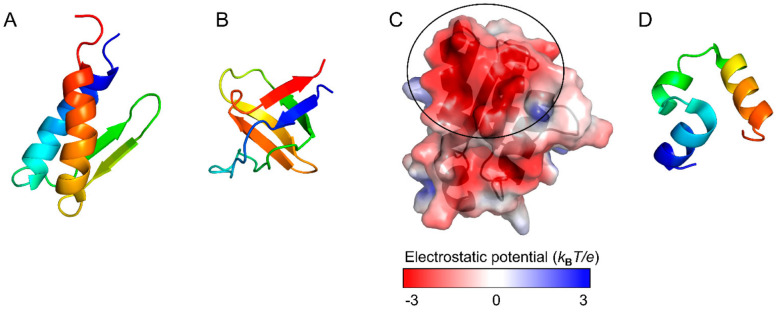
Native structures of gpW (PDB ID: 1HYW) (**A**) and the SH3 domain (PDB ID: 1SHG) (**B**). (**C**) Electrostatic potential energy surface of barstar (PDB ID: 1BTA). The black circle shows the barnase-binding site with large negative potentials. The electrostatic potential was calculated by APBS [76]. (**D**) Native structure of the 35-residue subdomain from the villin headpiece (PDB ID: 1YRF). Figures were drawn using PyMOL Molecular Graphics System, Version 2.4.0 Schrödinger, LLC.

Although two small proteins, gpW and the SH3 domain (Figure 4A,B), have comparable thermodynamic stability, experiments revealed that gpW folds ~1000-fold faster than SH3 [65]. Consistent with these observations, the WSME model with improved contact-energy calculations predict that the free-energy landscape of gpW has a marginal barrier, whereas that of SH3 has a clear barrier and exhibits cooperative two-state folding [65]. Similarly, the free-energy landscapes were compared between the WW domain of PIN1, a two-state folder, and BBL, which was experimentally shown to be a downhill folder without a clear free-energy barrier [51]. The WSME model predicts that PIN1 has a free-energy landscape with a distinct barrier, whereas BBL has an overall downhill landscape at low temperatures. Thus, the WSME model successfully explains the folding mechanisms of small proteins.

Remarkably, the WSME model proposes that the folding mechanism of BBL is temperature-dependent, involving a downhill folding in a biologically relevant temperature range and a barrier-limited cooperative folding with a slight free-energy barrier at the transition midpoint temperature (*T*_m_) [51,61]. This indicates that downhill and two-state folding mechanisms are continuously connected along temperatures and belong to the same folding class. The WSME model also successfully quantifies the free-energy barrier of PDD, a protein homologous to BBL [69,70,77]. The free-energy landscapes of PDD show downhill folding at low temperatures, but show two-state folding with a small free-energy barrier around the *T*_m_. Therefore, the WSME model can quantitatively characterize the temperature dependence of folding mechanisms, even for proteins with small free-energy barriers.

### 3.2. Two-Dimensional Free-Energy Landscape: Multiple Folding Pathways

**Figure 5:**
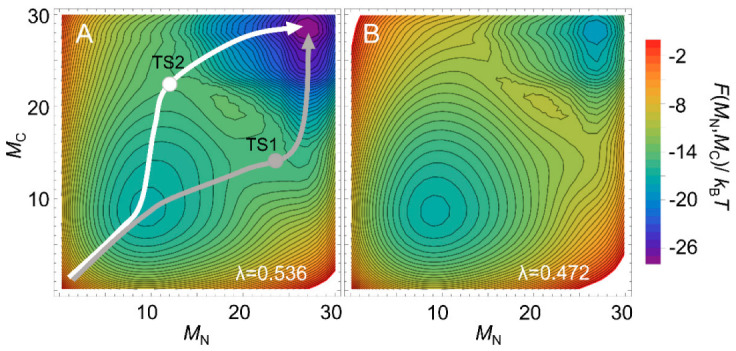
Two-dimensional free-energy landscapes of BdpA under folding conditions (**A**) and at the transition midpoint (**B**). The native structure of BdpA is shown in Figure 2 (left panel). *M_N_* and *M_C_* are the number of folded residues in the N-terminal half of BdpA (involving the first helix and the first half of the second helix) and C-terminal half of BdpA (involving the second half of the second helix and the third helix), respectively. Gray and white arrows denote the dominant folding pathways passing through the saddle points corresponding to the transition state 1 (TS1) and transition state 2 (TS2), respectively. *λ =* *ε*/(*k*_B_ *T*) is a parameter related to the uniform contact energy *ε* and temperature *T*. Adapted with permission from Ref. [26]. Copyright (2006) National Academy of Sciences, U.S.A.

Multi-dimensional representations of free-energy landscapes can be achieved using multiple reaction coordinates corresponding to the structural formation of multiple regions of a protein [78]. Such multi-dimensional free-energy landscapes allow the visualization of detailed folding pathways. Moreover, the WSME model can predict the degree of structure formation of each residue along a folding pathway [26,79,80,81]. Using the WSME model with uniform contact energies, Sasai et al. calculated the free-energy landscapes of the B domain of protein A (BdpA), consisting of three helices with a symmetric topology (Figure 2) [26]. The 1D free-energy profile indicates that BdpA folds in a two-state manner (Figure 2), and the 2D free-energy landscape identifies two major folding pathways (Figure 5A). These pathways were revealed for the first time by describing the multi-dimensional free-energy landscape. Previous experimental studies of BdpA folding using Φ-value analysis showed that the second helix is the most structured in the transition state [82]. However, MD simulations could not reproduce these observations. By contrast, the WSME model provides folding processes that are in agreement with experimental results. The model suggests that proteins with symmetrical structures, such as BdpA, have two nearly symmetrical folding pathways (Figure 5A) [26]. In the transition state of one pathway (TS1), the first and second helices of BdpA are partially formed, whereas in the transition state of the other pathway (TS2), the second and third helices are partially formed. When these pathways are averaged, the second helix is the most completely formed in the transition state of the BdpA, which is consistent with experimental results [26].

The above prediction for BdpA used uniform contact energies for all the native contacts, emphasizing the importance of a symmetric topology. Interestingly, the calculations predict that the two contrasting pathways will occur almost equally near room temperature (Figure 5A), whereas at higher temperatures, the symmetry is broken, and the folding is biased toward one pathway (Figure 5B). However, experimental Φ-values at a high temperature (near *T*_m_) did not verify this prediction [83]. Zamparo and Pelizzola examined the temperature dependence of the folding pathways of four proteins (BdpA, albumin-binding domain (ABD), designed α3D protein, and engrailed homeodomain) with similar folds consisting of three helices using contact energies weighted according to the number of atoms involved in the contact rather than uniform contact energies [52]. The results suggest that even for proteins with symmetric structures, the folding abilities of the N- and C-terminal regions depend on subtle differences in the native contacts involved, and the transition-state structure is almost independent of temperature, which is in agreement with the results of experiments. The results also highlight the importance of accurate contact energies for the reliable prediction of protein-folding pathways [52].

### 3.3. Effects of Amino-Acid Substitutions on Stability and Folding

Predicting protein stability is difficult because the 3D structures of proteins are only marginally stabilized by networks of weak non-covalent interactions. Thus, amino-acid substitutions in proteins can have complex effects on the free-energy landscapes, changing the free energies of the native and unfolded states, as well as the number and nature of folding intermediates. Nevertheless, as shown above, the WSME model has the potential to predict the effects of amino-acid substitutions on protein stability and folding by calculating the free-energy landscapes of wild-type and mutant proteins. Such calculations have provided useful insights for protein engineering and medical applications [22,44,64,84,85,86]. Naganathan et al. proposed a framework for calculating the stability of mutants using the WSME model and developed two programs, pStab [21] and pPerturb [22], which are available online. These methods may be useful as the first steps in screening protein mutants with the desired stability.

The relationship between folding and function has been examined by comparing the free-energy landscapes of homologous proteins with those of proteins with amino-acid substitutions or chemical modifications [62,63,74,80,87,88,89,90,91,92]. The charge distribution on the protein surface is one of the key factors controlling ligand binding and can also affect protein stability and folding [62,63,74]. For example, it has been suggested that barstar (Figure 4C) maintains its ability to bind to barnase by acquiring a large binding surface with negative electrostatic potential during evolution, resulting in a complex free-energy landscape with multiple folding intermediates [88]. Theoretical calculations predict that amino-acid substitutions to neutralize the charges at the barnase-binding site would improve the stability of barstar and simplify its folding mechanism to “frustration-free” two-state folding [88]. Thus, the WSME model may be useful for evaluating the effects of amino-acid substitutions and clarifying the role of each residue in the stability, folding, and function of proteins.

### 3.4. Effects of External Forces on Protein (un)Folding

An extended WSME model with external forces was constructed as a theoretical model of mechanical unfolding experiments on a single-protein molecule using atomic-force microscopy (AFM) [93]. The model calculates the equilibrium force-extension curves and free-energy landscapes as a function of the end-to-end length of a protein to characterize mechanical unfolding [93,94,95]. The kinetics of the response to time-dependent external forces (force clamp and dynamic loading) can also be evaluated by combining Monte Carlo simulations. Such analyses of the mechanical unfolding of ubiquitin predict the order of secondary structure formation and the presence of kinetic intermediates, which are consistent with the results from experiments and all-atom MD simulations [96]. In addition, this extended WSME model predicts the major and minor unfolding pathways of green fluorescent protein observed experimentally [97] and was further applied to characterize the equilibrium properties and kinetic unfolding pathways of RNAs, such as an RNA hairpin and the *Tetrahymena thermophila* ribozyme [98,99].

Single-molecule experiments with AFM have also shown that glycerol, a protective osmolyte, stabilizes the native state of globular proteins against mechanical unfolding without changing the position of the transition state on the reaction coordinate [100]. To simulate the mechanical unfolding of a protein in the presence of osmolytes, extended versions of the WSME model that consider the effects of osmolytes were developed [100,101]. The model successfully reproduces the experimental results of mechanical unfolding, in which the position of the transition state along the reaction coordinate is unchanged by osmolytes for the immunoglobulin-binding B1-domain of *Streptococcal* protein G (GB1) and the I27 module of human cardiac titin [100,101]. Thus, the WSME model with external forces is useful for understanding the single-molecule behavior of proteins during mechanical unfolding.

The external force term introduced in the above models has also been used to evaluate the effects of crowded environments, such as inside cells, on protein stability and folding. The predictions for ABD, GB1, and the β-hairpin of GB1 indicate that as the cage size confining a protein gradually decreases, the protein molecule will be stabilized up to a certain threshold cage size, and then destabilized below the threshold [102]. Furthermore, a general relationship between cage size and folding rate has been observed for various proteins [102]. A model for non-equilibrium diffusion dynamics was also developed using an external force term to describe the intracellular translocation of proteins [103]. Thus, the WSME model with external forces is also useful for theoretically evaluating protein stability and folding in various situations in which mechanical forces act on proteins.

### 3.5. Folding Kinetics and Transition State

The macroscopic kinetic behavior of protein states during folding can be predicted with kinetic models, such as master equations, using the free energies of the unfolded, intermediate, transition, and native states obtained from the WSME model. The theoretical folding rates thus obtained were shown to depend on the protein topology, which was consistent with experimental observations [13,37,50,51,104,105]. For example, the predicted folding rates of the 35-residue subdomain from the villin headpiece, which has three short α-helices (Figure 4D) and exhibits ultrafast folding, are consistent with those measured experimentally [38,39,40,41]. Thus, the WSME model is a powerful tool for studying subtle differences in folding rates [38,73,74,75,106]. Because virtual amino-acid substitutions can be introduced by perturbing specific contact energies, the WSME model with such perturbations can be used to calculate the theoretical Φ-values along the folding pathway [13,26,78,105,107,108,109,110]. Sasai et al. calculated theoretical Φ-values for the transition state in the folding of BdpA by averaging the transition-state structures on both of the major folding pathways (Figure 5A) and succeeded in obtaining Φ-values consistent with experiments [26]. Thus, theoretical Φ-value analysis using the WSME model can describe folding reactions at the resolution of individual residues.

The free-energy landscapes obtained by the WSME model can be combined with Monte Carlo simulations using the Metropolis algorithm to simulate single-molecule trajectories and examine microscopic protein-folding kinetics [42,45,53,65,71,72,87,88,106,111,112]. Since the WSME model is a Gō-type coarse-grained model with a limited number of possible conformations, simulations of protein-folding reactions can be performed with low computational complexity. An ensemble average of many single-molecule trajectories reproduces the macroscopic folding behaviors [51,61,65]. The use of such simulations for several proteins suggests that even proteins that exhibit simple two-state folding have a variety of folding pathways with different transition-state structures, and that the experimentally observed transition state is the average of these structures [72,110]. This method is expected to resolve the possible discrepancies between the experimental results and a small number of MD trajectories for protein folding/unfolding reactions, as it provides a rich dataset of single-molecule folding trajectories that cannot be obtained from MD simulations.

Although the spatial resolution of the WSME model is lower than that of all-atom MD simulations, it has been suggested that folding/unfolding simulations with the WSME model reproduce all-atom MD simulations [42]. A comparison of the folding/unfolding trajectories for the villin headpiece based on the WSME model using DSA/L with those of the long-time all-atom MD simulations using explicit solvent, performed by Shaw et al., shows that the folding behaviors are very similar in both simulations, including the rate of transition between relevant conformations and the order of helix formation [42]. Since the WSME model only considers the residue–residue interactions occurring in the native structure, these results highlight the importance of native contacts in determining protein-folding mechanisms.

## 4. Folding Mechanisms of Multi-Domain Proteins

In the previous sections, we showed that the folding mechanisms of small single-domain proteins are described well by the WSME model. By contrast, the folding mechanisms of multi-domain proteins have been less frequently studied because they have complex, multiple folding pathways and intermediates, making it difficult to theoretically predict the folding processes [53,75,78,81,91,106,108,112,113,114,115,116]. However, multi-domain proteins comprise the majority of the proteome, and more than 70% of eukaryotic proteins contain multiple domains [117,118]. Therefore, the elucidation of the folding mechanisms of multi-domain proteins is an important issue in the life sciences. The two major ways of connecting two globular domains are (1) the tandem connection of two domains by a linker and (2) the insertion of one domain into another domain.

### 4.1. Tandem Connection of Multiple Domains

**Figure 6:**
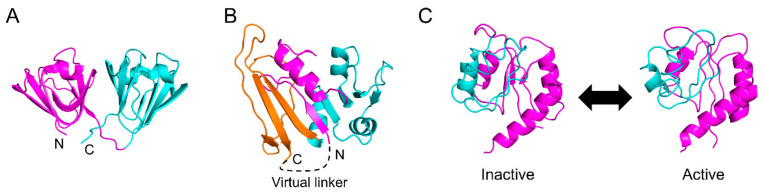
(**A**) Native structure of human γD-crystallin (PDB ID: 1HK0). Domains 1 and 2 are shown in magenta and cyan, respectively. (**B**) Native structure of *Escherichia coli* dihydrofolate reductase (DHFR) (PDB ID: 1RX1). The N- and C-terminal parts of the discontinuous loop subdomain (DLD) are shown in magenta and orange, respectively, and the adenosine-binding subdomain (ABD) is shown in cyan. In the extended WSME (eWSME) model, a virtual linker (dashed line) was implemented to virtually connect the N- and C- termini, both of which are included in the DLD. (**C**) Inactive and active conformations of nitrogen regulatory protein C (NtrC) (PDB ID: 1DC7 and 1DC8, respectively). Phosphorylation of NtrC induces allosteric conformational changes to the residues shown in cyan. Figures were drawn using PyMOL Molecular Graphics System, Version 2.4.0 Schrödinger, LLC.

The WSME model assumes that folding starts at local segments and then spreads throughout the molecule via the extensions and connections of the folded segments. Thus, the model is suitable for multi-domain proteins consisting of tandemly connected small globular domains, each of which folds in a two-state manner (Figure 6A). Typical examples are repeat proteins, and predictions of their folding processes are in agreement with experiments in terms of the structures of folding intermediates and the order of domain formation [53,75,78,113,114].

Sasai et al. applied the WSME model to multi-domain proteins with two globular domains connected in tandem, including γD-crystallin (Figure 6A), spore coat protein S, and R16-R17 spectrin domain, and investigated the effects of domain–domain interactions on folding reactions [108]. The computational results consistently explained the folding pathways and transition-state structures obtained by Φ-value analysis and suggested that the connectivity and interaction between the two domains determine the equilibrium and kinetic folding mechanisms. Furthermore, high-dimensional free-energy landscapes are effective in analyzing complex folding mechanisms and reveal hidden folding pathways, intermediates, and transition states for barnase, nitrogen regulatory protein C (NtrC), and an ankyrin repeat protein [78]. Although the computational complexity increases as the protein size increases, an efficient method to reduce computational complexity has been reported that considers short segments as blocks [114]. Note that when domain–domain interactions are strong in multi-domain proteins, the folding mechanisms may become more complex, making the prediction of folding processes more challenging, even for multi-domain proteins with tandem connections.

### 4.2. Domain Insertions

Another mode of domain connection is domain insertion. There are many multi-domain proteins in which one domain is inserted into another [119]. Many folding experiments have been performed on multi-domain proteins with domain insertions, including dihydrofolate reductase (DHFR), apomyoglobin, barnase, α-lactalbumin from bovine, human, and goat sources, and lysozyme from hen-egg-white, human, equine, and canine sources [2,4,5,9,30,36,54,55,57,59,60,120,121,122,123,124,125,126,127,128,129,130,131]. Interestingly, these proteins accumulate molten globule-like folding intermediates in which the discontinuous domain is more organized than the inserted continuous domain [5]. Such intermediates may be formed via a hydrophobic collapse mechanism driven by non-local hydrophobic interactions between distant residues in the amino-acid sequence [5,8,130]. The original WSME model cannot provide free-energy landscapes consistent with experiments for these proteins because it assumes that all the intervening domains must be folded before the discontinuous domain starts to fold.

DHFR is one of the most closely studied proteins in terms of its kinetic folding mechanism [5,120,122,123,127,128,129,130,131]. DHFR consists of two domains, with one globular domain (adenosine-binding subdomain, ABD) inserted into the other globular domain (discontinuous loop subdomain, DLD) (Figure 6B). We previously showed that the folding reaction of DHFR involves at least seven phases and six intermediates [131]. In brief, DHFR first forms a compact intermediate within 35 μs after the initiation of the folding reaction, and then DLD and ABD fold independently with time constants of 550 μs and 200 ms, respectively, accumulating an intermediate in which DLD is more organized than ABD. Finally, both domains dock to form the native structure. We also revealed that after a few milliseconds of folding, the folding behavior of “circular DHFR” with a disulfide bond introduced between the N- and C-termini is almost identical to that of “linear DHFR” with the disulfide bond reduced [128]. This suggests that the interactions between the N- and C-termini involved in DLD are already formed in the early stages of folding. However, these folding processes cannot be explained using the original WSME model.

To facilitate the folding of a discontinuous domain, Sasai et al. developed an extended WSME (eWSME) model, in which a virtual linker was introduced at the N- and C-termini of DHFR (Figure 6B) [45]. In this model, even when the inserted continuous domain (ABD) is not folded, non-local interactions can be formed between the N- and C-terminal regions involved in DLD via the virtual linker. The free-energy landscape calculated by the eWSME model successfully predicts the two of the six folding intermediates reported in the experiments [45,132]. Furthermore, Sasai et al. proposed that the introduction of multiple virtual linkers into a protein molecule may enable the prediction of the folding processes of multi-domain proteins with more than two domains [17]. Thus, the WSME model may be applicable for predicting the free-energy landscapes of a variety of multi-domain proteins after sufficient modifications. However, such modifications may not be easily implemented because it is not clear where and how many virtual linkers should be introduced in a protein molecule. Furthermore, as the number of virtual linkers increases, the mathematics describing them may become more complex. Nevertheless, the development of a modified version of the eWSME model that can be applied to any protein, including both small single-domain proteins and large multi-domain proteins, would represent significant progress toward solving the folding-process component of the “protein-folding problem” [12].

## 5. Applications beyond Protein Folding

### 5.1. Intrinsically Disordered Proteins

In addition to protein folding, the WSME model is also applicable to the conformational changes associated with protein function. Intrinsically disordered proteins (IDPs) have disordered structures in isolation but fold into specific structures upon binding to their partners [5,133,134,135]. For example, the intrinsically disordered region (IDR) of a neuron-restrictive silencer factor (NRSF) takes various β-hairpin-like structures in isolation but forms an α-helical structure when bound to its target protein, Sin3 [136,137,138]. Disordered structures of NRSF are theoretically created in the absence of Sin3 by introducing interactions favoring the β-hairpin structure into the WSME model; such interactions are different from those stabilizing the NRSF-Sin3 complex [136,137]. Furthermore, the free-energy landscape for the binding of NRSF to Sin3 obtained from this model reproduces the coupled folding and binding behaviors [136,138] commonly observed in many IDPs [5,133,134,135].

The free-energy landscape of an intrinsically disordered DNA-binding domain of the transcriptional regulator CytR, calculated using the WSME model, suggests that the conformational ensemble of the disordered state involves competition for several specific conformations [68]. By introducing the interaction between CytR and its partner DNA, the model successfully describes how, as the partner DNA approaches CytR, the free-energy landscape of CytR in the disordered state with multiple local minima changes into a landscape with a global minimum corresponding to the DNA-bound form of CytR [111,139]. Furthermore, the free-energy landscapes of CytR in the presence of a polymeric crowder, polyethylene glycol (PEG), mimicking crowded intracellular environments, provide a PEG concentration–temperature phase diagram showing that CytR is more folded both at lower temperatures and at higher PEG concentrations, which is in agreement with experimental results [140].

Thus, the WSME model comprehensively explains both the folding of globular proteins and the structures of IDPs in free and bound forms based on free-energy landscapes. In addition, the model can predict the effects of temperature, osmolytes, and amino-acid substitutions on IDP structures and may be useful for controlling the conformations of IDPs [141,142]. It may also be possible to predict the effects of ion valence and ionic strength on the free-energy landscapes of IDPs by incorporating them into the contact energies. The next target of IDP studies using the WSME model would be to predict the mechanisms of the coupled folding and binding reactions of IDPs [5,135].

### 5.2. Conformational Changes Associated with Protein Function

Many proteins dynamically change their conformations and exert their functions by binding to specific targets or through post-translational modifications. Free-energy calculations using the WSME model have also been applied to the theoretical analysis of the conformational changes associated with protein functions, such as photocycles and allosteric transitions [17,143,144,145,146].

Photoactive yellow protein (PYP) is a model protein for photoreceptors that has a photocycle consisting of three states [147]. The cycle involves coordinated motion on different time scales, from isomerization of chromophores, occurring in nanoseconds, to the partial denaturation of proteins, occurring in milliseconds or more [147]. Sasai et al. constructed an extended model describing motions over a wide range of time scales by adding an energy term to the WSME model that depends on local packing changes. The calculations assuming the ground state of the photocycle to be the native state yield a free-energy landscape that reasonably reproduces the photocycle and predicts the detailed structure of each state involved in the cycle [143,144]. Thus, the WSME model successfully explains the mechanism through which local structural fluctuations induce large-scale conformational changes, suggesting that the close interplay of motions at different time scales plays a crucial role in regulating protein function.

Sasai et al. further modified the WSME model to allow multiple native states and developed an allosteric WSME (aWSME) model that can calculate a free-energy landscape reflecting protein allostery [17,145,146]. The bacterial enhancer-binding protein, NtrC, undergoes an allosteric transition from the inactive to the active state through phosphorylation (Figure 6C). The application of the aWSME model to NtrC yields free-energy landscapes that predict large conformational fluctuations between the inactive and active states, as well as allosteric conformational changes upon phosphorylation [145]. The aWSME model also predicts that the GTP-binding protein Ras is stabilized by binding to GDP, whereas the structure of Ras in the GTP-bound state fluctuates significantly, suggesting that the difference in conformational fluctuations between the GDP- and GTP-bound states regulates signal transduction [146]. Thus, the aWSME model, which allows multiple native states, provides a mechanistic explanation for the transitions between multiple stable conformations and allosteric conformational changes upon effector binding.

Cnu is a transcriptional co-repressor that regulates gene expression upon temperature changes and has also been proposed to be involved in pH-dependent gene expression [148,149,150]. Using the WSME model with rigorous contact energy-calculations, including electrostatic interactions, Naganathan et al. showed that the distribution of conformations in the native-state ensemble of Cnu is sensitive to changes in both temperature and pH, suggesting that Cnu can serve as both a temperature and a pH sensor [148,149,150].

### 5.3. Other Applications

Amyloids are insoluble fibrous aggregates of proteins stabilized primarily by hydrogen bonds and hydrophobic interactions and have a cross β-sheet structure with parallel β-strands aligned perpendicular to the fibril axis [151,152]. Because amyloids are implicated in neurodegenerative diseases, such as Alzheimer’s disease, Parkinson’s disease, and bovine spongiform encephalopathy, understanding the mechanisms of amyloid fibril formation is an important issue in drug discovery [151,152]. The assumption of the WSME model, in which an entire protein molecule folds through the elongation and docking of local native segments, has also been utilized as a model to describe amyloid formation [153,154]. By introducing both the interactions stabilizing the monomeric form of a protein and those stabilizing the amyloid form, the WSME model was able to qualitatively reproduce a sharp phase transition to amyloid fibrils, which is characteristic of the nucleation-growth model and is consistent with experiments on amyloid formation [153,154].

Since the WSME model can be regarded as a simple 1D lattice model, the exact solution of the model can be calculated using the transfer matrix method, even for systems with non-uniform interactions. Taking advantage of this feature, the WSME model has also been used to describe the growth of strained epitaxy [155,156]. Furthermore, the WSME model itself has been a subject of research in theoretical statistical mechanics, and efforts have been made to develop kinetic analyses by applying the cluster-variation method, which is one of the most precise methods for solving the Ising model when an exact solution is not available [50,157,158,159]. Other studies have examined the relationship between protein structures and folding mechanisms through the partition function zeros of the WSME model [160,161,162,163]; partition functions for various secondary-structure elements and two small proteins (BBL and chymotrypsin inhibitor 2) have shown that the distribution of partition function zeros distinguishes folding mechanisms, such as downhill and two-state folding [161].

## 6. Summary and Future Perspectives

In this review, we summarized how the WSME model and its extended versions describe protein folding and dynamics. The WSME model can calculate the free-energy landscapes of proteins, which predict the thermodynamic quantities involved in equilibrium-unfolding transitions and the pathways and structures involved in kinetic folding processes. These calculations are consistent with the experimental results of protein folding, especially for small single-domain proteins, suggesting that the WSME model enables the prediction of detailed protein-folding processes that are difficult to measure experimentally, and contributes to our understanding of protein-folding mechanisms. Surprisingly, although the WSME model is a simple coarse-grained model, it can reproduce various aspects of protein folding obtained by all-atom MD simulations. This agreement strongly supports the hypothesis that folding reactions are primarily driven by native interactions and that the free-energy landscape is globally biased toward the native state. This also indicates that the WSME model adequately captures and deciphers the bias encoded in protein conformation. Therefore, the WSME model, when combined with rigorous contact-energy calculations, provides theoretical predictions that are in good agreement with the experimental results for small proteins.

The WSME model has also been applied to predict the folding mechanisms of multi-domain proteins, especially those consisting of tandemly connected small globular domains. Although it is difficult to compute entire folding reactions of large multi-domain proteins using all-atom MD simulations, the WSME model can calculate the free-energy landscapes of such proteins with low computational complexity. Therefore, the WSME model and MD simulations are expected to be important tools for predicting protein-folding mechanisms.

Nevertheless, it is still challenging to predict the folding mechanisms of proteins with complex structures, such as multi-domain proteins with domain insertions and those with strong interactions between domains. Although non-local interactions between distant segments in the amino-acid sequence may be formed early in folding reactions by the hydrophobic collapse mechanism [5], they cannot be considered in the original WSME model. One promising approach to solving this problem is to introduce virtual linkers at non-local contacts that can be formed early in the folding reaction. Indeed, introducing a single virtual linker between the N- and C-termini is effective in predicting the folding processes of DHFR [17,45]. The next challenge would be to introduce multiple virtual linkers at arbitrary positions in a single protein to enable the prediction of the folding mechanisms of any protein, including small single-domain proteins and large multi-domain proteins with complex main-chain topologies.

Another type of interaction that complicates the protein structure is a disulfide bond. The WSME model has never explicitly considered the folding reactions of disulfide-intact proteins or those involving oxidative formation of disulfide bonds. The prediction of such folding reactions is also challenging, but it may be achieved by replacing the virtual linkers introduced above as non-local interactions with covalent linkers.

Because of its simplicity and versatility, the WSME model can be used to analyze various biological events other than protein folding under equilibrium and non-equilibrium conditions by calculating free-energy landscapes using exact or approximate solutions and, subsequently, performing Monte Carlo simulations. Due to this utility, the extended version of the WSME model provides reasonable predictions for protein-conformation changes in IDPs and allosteric conformational changes accompanied by protein functions, such as protein–protein interactions and ligand binding. Furthermore, the model may be applicable to multimer formation, domain swapping, and the coupled folding and binding reactions of IDPs.

Recently, protein-structure prediction has made great advances, through deep-learning approaches, towards solving the structure-prediction component of the “protein-folding problem” [12,164]. However, even state-of-the-art structure prediction methods do not provide an understanding of how proteins fold into specific structures [165]. Therefore, the theoretical prediction of protein-folding processes remains a challenge. Since the WSME model can predict protein folding and dynamics with low computational complexity, the WSME model and its modifications will play an important role in solving the folding-process component of the “protein-folding problem” in the near future.

## Data Availability

Not applicable.
